# Link between Peripheral Artery Disease and Heart Rate Variability in Hemodialysis Patients

**DOI:** 10.1371/journal.pone.0120459

**Published:** 2015-08-03

**Authors:** Szu-Chia Chen, Chien-Fu Chen, Jiun-Chi Huang, Mei-Yueh Lee, Jui-Hsin Chen, Jer-Ming Chang, Shang-Jyh Hwang, Hung-Chun Chen

**Affiliations:** 1 Division of Nephrology, Kaohsiung Medical University Hospital, Kaohsiung Medical University, Kaohsiung, Taiwan; 2 Division of Neurology, Kaohsiung Medical University Hospital, Kaohsiung Medical University, Kaohsiung, Taiwan; 3 Division of Endocrinology and Metabolism, Department of Internal Medicine, Kaohsiung Medical University Hospital, Kaohsiung Medical University, Kaohsiung, Taiwan; 4 Graduate Institute of Clinical Medicine, College of Medicine, Kaohsiung Medical University, Kaohsiung, Taiwan; 5 Department of Internal Medicine, Kaohsiung Municipal Hsiao-Kang Hospital, Kaohsiung Medical University, Kaohsiung, Taiwan; 6 Department of Nursing, Kaohsiung Municipal Hsiao-Kang Hospital, Kaohsiung Medical University, Kaohsiung, Taiwan; 7 Faculty of Medicine, College of Medicine, Kaohsiung Medical University, Kaohsiung, Taiwan; Université de Montréal, CANADA

## Abstract

Peripheral artery disease (PAD) and low heart rate variability (HRV) are highly prevalent in hemodialysis patients, and both are associated with increased cardiovascular morbidity and mortality. This study aims to examine the suggested relationship between PAD and HRV, and the relationship of parameters before and after hemodialysis. This study enrolled 161 maintenance hemodialysis patients. PAD was defined as ABI < 0.9 in either leg. HRV was performed to assess changes before and after hemodialysis. The change in HRV (△HRV) was defined as post-hemodialysis HRV minus pre-hemodialysis HRV. Patients’ clinical parameters were collected from the dialysis records. All HRV parameters except high frequency (HF) % were lower in patients with PAD than patients without PAD, though not achieving significant level. In patients without PAD, HF (*P* = 0.013), low frequency (LF) % (*P* = 0.028) and LF/HF (*P* = 0.034) were significantly elevated after hemodialysis, whereas no significant HRV parameters change was noted in patients with PAD. Serum intact parathyroid hormone was independently associated with △HF (β = -0.970, *P* = 0.032) and △LF% (β = -12.609, *P* = 0.049). Uric acid level (β = -0.154, *P* = 0.027) was negatively associated with △LF/HF in patients without PAD. Our results demonstrated that some of the HRV parameters were significantly increased after hemodialysis in patients without PAD, but not in patients with PAD, reflecting a state of impaired sympatho-vagal equilibrium. Severity of secondary hyperparathyroidism and hyperuricemia contributed to lesser HRV parameters increase after hemodialysis in patients without PAD.

## Introduction

The incidence of non-traumatic lower extremity amputation among the end-stage renal disease (ESRD) population is higher than that among non-ESRD patients [1], and peripheral artery disease (PAD) is the most common indication for amputation [2]. PAD is also associated with increased cardiovascular mortality, morbidity and hospitalization in patients with ESRD [3]. In patients with ESRD and PAD, cardiovascular autonomic dysfunction and the related risk of arrhythmia may partially explain the observed high rate of cardiovascular mortality, besides the presence of coronary and cerebral atherosclerosis [4, 5]. In addition, low heart rate variability (HRV) has been recognized to associate with adverse cardiovascular outcomes in chronic hemodialysis (HD) patients [4, 5].

Cardiovascular autonomic neuropathy can be evaluated by HRV, a measure of variations in heart rate. In practice, it is defined as variations of both instantaneous heart rate and R-R intervals of electrocardiogram, and may provide a simple and noninvasive way in assessing the activities of the autonomic nervous system [6]. Abnormalities of HRV primarily reflect the dys-regulation between the sympathetic or parasympathetic nervous system. Frequency-domain analysis of HRV has gained popularity with broad application as a functional indicator of the autonomic nervous system, because of the non-invasiveness and easy accessibility. HRV has been categorized into high-frequency (HF) and low-frequency (LF) ranges [6]. HF is equivalent to the well-known respiratory sinus arrhythmia and is considered to represent vagal control of heart rate [7]. Both vagal and sympathetic activities jointly contribute to LF HRV [8]. Normalized LF (LF%) and the ratio LF/HF are considered to mirror the sympatho-vagal balance or to reflect sympathetic modulations [6]. A previous study reported that PAD patients showed increased sympathetic and parasympathetic modulation in the heart [9]. Diabetic patients with PAD had a higher level of dysfunction in autonomic modulation than diabetic patients without PAD [10].

Previous studies have shown that decreased HRV is prevalent among HD patients [4]. Furthermore, reduced HRV has been shown to be associated with adverse cardiovascular outcomes and mortality in ESRD [4, 5]. Patients with ESRD have been demonstrated to have changes in HRV during HD treatment. As HRV changes during a single dialysis session, autonomic function appears to improve with dialysis therapy [11, 12]. However, little is known about the association of PAD with the autonomic regulation in HD patients. In this study, we examined the relationship between PAD and HRV, and the relationship between PAD and HRV parameters before and after HD management.

## Subjects and Methods

### Study patients and design

The study was conducted in a regional hospital in southern Taiwan. All maintenance HD patients in this hospital were included, except patients receiving HD at night shift, and refusing ankle-brachial index (ABI) exams. Finally, we enrolled 161 patients (71 males and 90 females) from May 2012 to July 2012. All patients received HD three times per week, and each HD session was performed for 3.5–4.5 hours with a blood flow rate of 250–300 mL/min and dialysate flow of 500 mL/min. Blood samples were taken before HD, and also after HD to calculate Kt/V.

### Ethics statement

The study protocol was approved by the Institutional Review Board of the Kaohsiung Medical University Hospital (KMUH-IRB-20110445). Informed consents were obtained in written form from patients and all clinical investigation was conducted according to the principles expressed in the Declaration of Helsinki. The patients gave consent for the publication of the clinical details.

### Assessment of ABI and brachial-ankle pulse wave velocity (baPWV)

The values of ABI and baPWV were measured 10 minutes before HD. The ABI and baPWV were measured by an ABI-form device, which automatically and simultaneously measured blood pressures in both arms and ankles using an oscillometric method [13]. Occlusion and monitoring cuffs were placed tightly around the upper arm not harboring blood access and both sides of the lower extremities in the supine position. ABI was calculated by the ratio of the ankle systolic blood pressure divided by the arm systolic blood pressure, while the lower value of the ankle systolic blood pressure was used for the calculation. For measuring baPWV, pulse waves obtained from the brachial and tibial arteries were recorded simultaneously and the transmission time, which was defined as the time interval between the initial increase in brachial and tibial waveforms, was determined. The transmission distance from the arm to each ankle was calculated according to body height. The baPWV value was automatically computed as the transmission distance divided by the transmission time. After obtaining bilateral baPWV values, the higher one was used for further analysis. PAD was defined as ABI < 0.9 in either leg.

### Electrocardiogram signal processing

All recruited subjects received a short-term power spectral analysis of HRV. All measurements for spectral analysis were conducted in a quiet, temperature-controlled (28°C) room. The procedure for HRV analysis was designed according to the standard method, and detailed procedures for HRV analysis have been reported previously [14–16]. A pericardial electrocardiogram (ECG) was taken for continuous 5 min with the patients laying quietly and breathing normally in the supine position for at least 10 min. The study patients received ECG examination before and after HD 30 minutes, respectively, during the day (between 8 a.m. and 5 p.m.). ECG signals were recorded using an HRV analyzer (SS1C, Enjoy Research, Taipei, Taiwan) with an analog-to-digital converter and sampling rate of 256 Hz. Digitized ECG signals were analyzed online and were simultaneously stored for off-line verification. Signal acquisition, storage, and processing were performed on a computer. The computer algorithm then identified each QRS complex and rejected each ventricular premature complex or noise according to its likelihood in a standard QRS template. Stationary R-R values were re-sampled and interpolated at a rate of 7.11 Hz to produce continuity in the time domain [15].

### HRV frequency-domain analysis

Frequency-domain analysis was performed using a non-parametric method of fast Fourier transformation (FFT). The direct current component was deleted, and a Hamming window was used to attenuate the leakage effect [17]. For each time segment (288 s; 2048 data points), our algorithm estimated the power spectrum density based on FFT. The resulting power spectrum was corrected for attenuation resulting from the sampling and the Hamming window. The power spectrum was subsequently quantified into standard frequency-domain measurements as defined previously [6], including HF (0.15–0.40 Hz), LF (0.04–0.15 Hz) and LF/HF HRV. LF was normalized by the percentage of total power to detect the sympathetic influence on HRV (LF% = LF/(total power-very low frequency [VLF])*100). A similar procedure was applied to HF (HF% = HF/(total power-VLF)*100). All HRV parameters were logarithmically transformed to correct for the skewness of the distribution [6].

### Collection of demographic, medical, and laboratory data

Demographic and medical data including age, gender and co-morbid conditions were obtained from medical records and interviews with patients. The body mass index was calculated as the ratio of weight in kilograms divided by the square of height in meters. Laboratory data were measured from fasting blood samples using an autoanalyzer (Roche Diagnostics GmbH, D-68298 Mannheim COBAS Integra 400). Serum intact PTH (iPTH) concentration was evaluated using a commercially available two-sided immunoradiometric assay (CIS bio international, France). Kt/V was evaluated monthly as a marker of dialysis efficiency and was determined according to the Gotch procedure [18].

### Statistical analysis

Statistical analysis was performed using SPSS 15.0 for windows (SPSS Inc. Chicago, USA). Data are expressed as percentages, mean ± standard deviation, or mean ± standard error of the mean for HRV parameters, or median (25^th^-75^th^ percentile) for duration of dialysis, triglyceride and iPTH. The differences between groups were checked by Chi-square test for categorical variables, by independent t-test for continuous variables with approximately-normal distribution, or by Mann-Whitney U test for continuous variables with skewed distribution. Paired t-test was used to compare HRV parameters before and after HD. To examine further whether PAD was associated with HRV parameters change, generalized estimating equations (GEE) were used in multivariate analysis adjustment for diabetes mellitus (DM) and cerebrovascular disease. The ΔHRV parameters were defined as HRV parameters measured after HD minus HRV parameters measured before HD. Multiple forward linear regression analysis was used to identify the factors associated with ΔHRV parameters. A difference was considered significant if the *P* value was less than 0.05.

## Results

The mean age of the 161 patients was 61.5 ± 11.2 years. The prevalence of PAD was 31.1%. The comparison of baseline characteristics between patients with and without PAD was shown in Table 1. Compared with patients without PAD, patients with PAD were found to have an older age, higher prevalence of DM, higher prevalence of a history of cerebrovascular disease, lower creatinine, and lower uric acid. Except HF%, all the other HRV parameters were lower in patients with PAD than patients without PAD, though not achieving statistical significance.

**Table 1:** Comparison of baseline characteristics between patients with and without PAD.

Characteristics	All patients (n = 161)	Without PAD (n = 111)	With PAD (n = 50)	*P*
Age (year)	61.5 ± 11.2	60.1 ± 11.8	64.5 ± 9.3	0.021
Male gender (%)	44.1	45.9	40.0	0.482
Duration of dialysis (years)	5.9 (2.5–10.2)	6.0 (2.7–10.5)	5.5 (2.3–10.0)	0.740
Diabetes mellitus (%)	44.7	35.1	66.0	< 0.001
Hypertension (%)	62.7	65.8	56.0	0.236
Coronary artery disease (%)	25.5	23.4	30.0	0.375
Cerebrovascular disease (%)	14.9	10.8	24.0	0.030
Systolic blood pressure (mmHg)	156.3 ± 26.5	155.1 ± 24.4	158.9 ± 30.8	0.438
Diastolic blood pressure (mmHg)	81.7 ± 15.2	83.0 ± 14.9	78.8 ± 15.7	0.103
Body mass index (kg/m^2^)	23.9 ± 3.4	23.7 ± 3.2	24.3 ± 3.7	0.334
baPWV (cm/s)	1967.1 ± 567.6	1962.4 ± 516.1	1978.0 ± 680.6	0.877
Laboratory parameters				
Albumin (g/dL)	3.8 ± 0.3	3.9 ± 0.3	3.8 ± 0.3	0.050
Fasting glucose (mg/dL)	118.3 ± 48.5	113.8 ± 43.5	128.3 ± 57.2	0.118
Triglyceride (mg/dL)	127 (93–212)	124 (90–194)	148.5 (95.8–243.5)	0.068
Total cholesterol (mg/dL)	181.5 ± 43.0	182.9 ± 41.5	178.6 ± 46.3	0.559
Hemoglobin (g/dL)	10.2 ± 1.2	10.2 ± 1.2	10.1 ± 0.9	0.324
Creatinine (mg/dL)	9.7 ± 2.2	10.1 ± 2.2	9.0 ± 1.9	0.004
CaXP product (mg^2^/dL^2^)	41.7 ± 11.1	41.8 ± 11.2	41.5 ± 11.1	0.873
iPTH (pg/mL)	343.7 (183.8–487)	353.6 (183.7–483)	333.8 (188.6–536.5)	0.669
Uric acid (mg/dL)	7.8 ± 1.8	8.0 ± 1.8	7.3 ± 1.6	0.041
Kt/V (Gotch)	1.3 ± 0.2	1.3 ± 0.2	1.3 ± 0.2	0.863
Ultrafiltration (%)	4.1 ± 1.6	4.2 ± 1.4	3.9 ± 1.9	0.229
HRV parameters (frequency domain) before hemodialysis				
LF (ms^2^)	2.2 ± 0.3	2.5 ± 0.4	1.6 ± 0.6	0.157
HF (ms^2^)	2.1 ± 0.4	2.3 ± 0.4	1.6 ± 0.7	0.377
LF% (nu)	40.1 ± 1.8	42.2 ± 2.2	36.3 ± 3.1	0.074
HF% (nu)	33.1 ± 1.3	33.0 ± 1.6	33.2 ± 1.9	0.944
LF/HF	0.13 ± 0.10	0.22 ± 0.12	-0.07 ± 0.17	0.178

Abbreviations. PAD, peripheral artery disease; baPWV, brachial-ankle pulse wave velocity; CaXP product, Calcium-phosphorous product; iPTH, intact parathyroid hormone; HRV, heart rate variability; LF, low frequency; HF, high frequency.

### Change of HRV parameters before and after HD

HRV parameters changes before and after HD were shown in Table 2. In patients without PAD, HF (*P* = 0.013), LF% (*P* = 0.028) and LF/HF (*P* = 0.034) significantly increased after HD, whereas no significant change of HRV parameters was noted in patients with PAD.

**Table 2:** HRV parameters of patients with and without PAD before and after hemodialysis.

HRV parameters (frequency domain)	Without PAD		With PAD	
	Before hemodialysis	After hemodialysis	Before hemodialysis	After hemodialysis
LF (ms^2^)	2.5 ± 0.4	2.9 ± 0.5	1.6 ± 0.6	2.7 ± 0.6
HF (ms^2^)	2.3 ± 0.4	2.7 ± 0.4*	1.6 ± 0.7	2.5 ± 0.7
LF% (nu)	42.2 ± 2.2	47.0 ± 2.0*	35.3 ± 3.	40.4 ± 3.4
HF% (nu)	33.0 ± 1.6	30.3 ± 1.3	33.2 ± 1.9	30.2 ± 1.9
LF/HF	0.22± 0.12	0.46 ± 0.10*	-0.07 ± 0.17	0.16 ± 0.16

*P < 0.05 compared to patients before hemodialysis.

Abbreviations are the same as in Table 1.

We further performed GEE in multivariate analysis after adjustment for DM and cerebrovascular disease because both might influence the autonomic nervous system. The main effects of PAD on HF, LF% and LF/HF change by GEE revealed that patients without PAD were associated with an increase in LF% (*P* = 0.011) and LF/HF (*P* = 0.033), independent of DM and cerebrovascular disease, bur not HF (*P* = 0.288).

### Determinants of ΔHRV parameters in patients without PAD

Table 3 displayed the un-standardized coefficient β of ΔHRV parameters, significant in Table 2 in patients without PAD, after adjustment for age, sex, duration of HD, a history of diabetes, hypertension, coronary artery disease and cerebrovascular disease, systolic and diastolic blood pressure, body mass index, baPWV, albumin, fasting glucose, triglyceride, total cholesterol, hemoglobin, creatinine, calcium-phosphorous product, iPTH, uric acid, Kt/V and ultrafiltration percent. In the multivariate forward analysis, iPTH was independently associated with ΔHF (β = -0.970, *P* = 0.032) and ΔLF% (β = -12.609, *P* = 0.049). Besides, uric acid (β = -0.154, *P* = 0.027) was negatively associated with ΔLF/HF in patients without PAD.

**Table 3:** Determinants of ΔHRV parameters of patients without PAD.

ΔHRV parameters	Multivariate (Forward)	
	Unstandardized coefficient β (95% CI)	*P*
ΔHF		
PTH (log per 1 pg/mL)	-0.970 (-1.856, -0.084)	0.032
ΔLF %		
PTH (log per 1 pg/mL)	-12.609 (-25.165, -0.054)	0.049
ΔLF/HF		
uric acid (per 1 mg/dL)	-0.154 (-0.291, -0.018)	0.027

Values expressed as unstandardized coefficient β and 95% confidence interval (CI). Abbreviations are the same as in Table 1.

Covariates in the multivariate model included age, sex, duration of dialysis, a history of diabetes, hypertension, coronary artery disease and cerebrovascular disease, systolic and diastolic blood pressure, body mass index, baPWV, albumin, fasting glucose, triglyceride, total cholesterol, hemoglobin, creatinine, CaXP product, iPTH, uric acid, Kt/V and ultrafiltration percent.

## Discussion

In the present study, we evaluated the relationship between PAD and HRV in HD patients. We found that HRV parameters, except HF%, were lower in patients with PAD than patients without PAD, although the difference did not achieve significant level. In addition, HF, LF% and LF/HF significantly increased after HD in patients without PAD, whereas no HRV parameters increased significantly in patients with PAD. Increased iPTH and uric acid were correlated with HRV parameters changes before and after HD in patients without PAD.

PAD is more prevalent in ESRD patients than in the general population, and is closely related to the higher morbidity and mortality [19–23]. For patients with atherosclerosis and PAD, ABI was recognized to be a good diagnostic tool and clinical marker. An ABI <0.9 has been utilized to identify peripheral artery occlusive disease both in epidemiologic studies and clinical practice [20]. HD patients having abnormally low ABI had worse prognosis for all-cause and cardiovascular mortality [24]. In PAD, sustained impairment of microcirculatory perfusion persistently impairs arteriolar vasodilator capacity, capillary perfusion, and the endothelial function [25, 26]. Endothelial dysfunction has been associated with impaired autonomic function, and increasing evidence has demonstrated that sympathetic neural control is involved in the vasomotor control of both and large small resistance arteries [27]. In humans, large artery stiffness has been associated with increased sympathetic discharge, both in healthy subjects and in renal transplant recipients. Peripheral sympathetic discharge is also able to modulate wave reflection. On the other hand, large artery stiffness can interfere with autonomic regulation by impairing carotid baroreflex sensitivity [27]. Interactions and the reciprocal influences between arterial stiffness and impaired autonomic regulation may, therefore, contribute significantly to the high prevalence of cardiovascular morbidity in HD patients.

HRV is a non-invasive measure of autonomic nervous system, which reflects beat-to-beat variability in heart rate, and has been successfully applied in chronic dialysis patients [28]. Canani et al. had evaluated the association between cardiovascular autonomic dysfunction and PAD in patients with type 2 DM, and found that patients with PAD had lower HRV parameters than patients without PAD [10]. In contrast, Goering et al. found higher HRV parameters in cardiovascular patients with PAD than patients without PAD, suggesting elevated vagal and sympathetic activation in cardiovascular patients with PAD [9]. Longenecker et al. had also investigated the association between HRV and atherosclerotic cardiovascular disease in 115 chronic HD patients. They found low HRV parameters were strongly associated with prevalent atherosclerotic cardiovascular disease [29]. In our study, HRV parameters were lower in patients with PAD, defined as ABI < 0.9, than patients without PAD, although not achieving significant level. Almost half of our patients (44.7%) were diabetic and 22.5% had pre-existed and documented coronary artery disease. Therefore, the results of HRV in our patients may be mediated by the interaction of insulin resistance and autonomic activation, and resulted in not statistically significant difference between patients with PAD and without PAD.

Dialysis-induced changes in autonomic cardiovascular modulation showed comparable findings using HRV in HD patients. Barnas et al. evaluated the changes in autonomic nervous system during HD and ultrafiltration. They found an increase in LF component of HRV during non-hypotensive dialysis in 26 HD patients. The change agreed with compensatory baroreflex-mediated activation of the sympathetic nervous system [30]. Zitt E et al. also evaluated the association of diabetes on autonomic cardiovascular regulation during HD. In contrast to 8 diabetic patients who showed a blunted autonomic response, in 9 non-diabetic patients, LF, HF and LF/HF increased during dialysis [31]. Impaired autonomic function might be related with diabetic damage in autonomic neuropathy. We also found some HRV parameters (HF, LF%, LF/HF) increased after HD in patients without PAD, but not in patients with PAD. Changes in HRV can be interpreted as compensatory mechanisms for vascular arteriolar vasodilator capacity in patients without PAD, but diminished in patients with PAD with impaired sympatho-vagal equilibrium. We propose that in PAD patients, the obviously diminished compensatory increase in sympathetic and parasympathetic activity *per se* might enhance the risk for hemodynamic instability, and further connected to cardiovascular diseases.

Secondary hyperparathyroidism and the calcium/phosphorous dys-regulation had been reported to contribute to endothelial dysfunction and vascular calcification in HD patients [32, 33]. Elevated parathyroid hormone (PTH) contributes to cardiovascular calcification, diminished cardiac contractility, and enhanced coronary risk [34, 35]. PTH also plays a role in disturbances in both sympathetic and parasympathetic functions. Polak et al. found lower LF and HF in 40 HD patients with high serum levels of iPTH, which indicated deterioration in total autonomic activity [36]. Zhang et al. had evaluated the relationship between mineral metabolism and HRV in chronic kidney disease stage 5 patients. They found that abnormal serum levels of iPTH, calcium and phosphorous were significantly associated with low HRV parameters, and parathyroidectomy might reverse the cardiovascular risk [37]. These results suggested that both dys-regulated cardiovascular autonomic control and abnormal mineral metabolism might participate in the pathogenesis leading to the higher risk of cardiovascular disease. We also found that higher iPTH level was associated with less HRV parameters increase in patients without PAD. In addition, our results also showed an association between hyperuricemia and less HRV parameters increase in patients without PAD. Previous studies had reported that hyperuricemia was associated with low HRV parameters [38, 39]. Hyperuricemia is affected by risk factors linked to insulin resistance, such as obesity, dyslipidemia and hypertension [40–42]. The proposed association between hyperuricemia and autonomic dysfunction could be explained by the commonly seen insulin resistance in HD patients, which has been shown to be correlated with low HRV [43]. Lowering PTH and uric acid levels might, therefore, improve HRV increase in HD patients, but future studies are needed to confirm such a proposition.

There are certain limitations in this study. First, this study was cross-sectional, and the causal relationship could not be confirmed. Future prospective studies would be needed to address this issue. Further, there is a circadian pattern of heart rate autonomic modulation with a reduced HRV during the day because of increased sympathetic activity, and an increased HRV during the night due to the predominance of vagal modulation [44, 45]. In our study, we performed all HRV examinations during the day (between 8 a.m. and 5 p.m.) to minimize the influence of circadian rhythm, but we could not eliminate the possibility that the rhythm might have been lost in some patients and the influence could not be defined. Perhaps a longer ECG record would help establish a baseline for each patient. Finally, HRV could be measured in the time or frequency domain. In our study, there was no time-domain measure. Although frequency-domain parameters correlated well (*r* = 0.85) with time-domain parameters, several time-domain parameters had proven to be useful for clinical purposes [46, 47]. Thus time-domain measures needed to be included in future studies. Furthermore, more recent measures of HRV (e.g. entropy-based measures, complexity/scale invariant/fractal measures) should be also investigated as spectral HRV measures have many limitations and their physiological interpretation is not as clear as previously thought.

In conclusion, our results showed that some HRV parameters significantly increased after HD in patients without PAD, but failed to rise in patients with PAD. It demonstrated a complex association between the impaired sympatho-vagal equilibrium and PAD. Secondary hyperparathyroidism and hyperuricemia contributed to less HRV parameters increase before and after HD in patients without PAD. We surely need further prospective follow-up and analysis to prove the clinical validity of PAD and HRV on patients’ outcomes, and also if reduction of PTH and uric acid would improve HRV parameters.
